# What Contributes to COVID-19 Vaccine Hesitancy? A Systematic Review of the Psychological Factors Associated with COVID-19 Vaccine Hesitancy

**DOI:** 10.3390/vaccines10111777

**Published:** 2022-10-22

**Authors:** John Romate, Eslavath Rajkumar, Aswathy Gopi, John Abraham, John Rages, R. Lakshmi, Joshy Jesline, Sudha Bhogle

**Affiliations:** 1 Department of Psychology, Central University of Karnataka, Kalaburagi 585367, India; 2 St. John’s Medical College, St. John’s National Academy of Health Sciences, Bangalore 560034, India; 3 Government College of Teacher Education, Calicut 673004, India; 4 Psychology & Head, Student Solutions, Eduquity Career Technologies, Bangalore 560095, India

**Keywords:** vaccine hesitancy, COVID-19, pandemic, psychological factors, systematic review

## Abstract

Vaccine hesitancy plays a crucial role in worldwide pandemic-control efforts. The multifaceted nature of vaccine hesitancy entails many psychological factors that are widely discussed in the literature, although few studies specifically compile these factors. Thus, this systematic review aims to synthesize the psychological factors contributing to vaccine hesitancy. As per the PRISMA (preferred reporting items for systematic reviews and meta-analyses) guidelines, a systematic search was conducted on electronic databases PubMed, Scopus, Science Direct, PsycNET, and Web of Science, and a manual search was conducted on Google Scholar. Out of the 2289 articles obtained, 79 studies that met the inclusion criteria were deemed eligible for the review. The findings highlight appraisals of the COVID-19 pandemic, vaccine safety and side effects, vaccine confidence/trust, trust in government and healthcare professionals, scepticism around vaccine production, conspiracy beliefs, emotions, and information and knowledge about the vaccine as the major psychological factors contributing to vaccine hesitancy. Concerningly, misinformation on COVID-19 vaccination spread through social media platforms, increasing vaccine hesitancy. Recommendations for government authorities, healthcare professionals, and implications for future research are also outlined.

## 1. Introduction

Sporadic outbreaks of contagious diseases have had a significant and long-lasting impact on societies throughout history. Vaccination has emerged as a critical healthcare response to the rising number of communicable diseases infecting the global population [1]. Even though a growing body of evidence reveals that vaccines are safe [2,3], vaccine hesitancy is also on the rise [4]. Vaccine hesitancy alludes to a lag in acceptance or refusal to uptake a vaccine despite the available facilities of vaccination programmes [2]. Further, the Sage Working Group has proposed that attitudes toward vaccination are influenced by three primary categories of variables: convenience, complacency, and confidence. Convenience pertains to vaccination accessibility, complacency refers to infection risk and immunization relevance, and confidence refers to belief in vaccine safety or efficacy [5].

Previous research has indicated vaccination hesitancy as a global issue, with many reasons for vaccine refusal [6,7]. Studies have explored hesitancy in cases of diseases such as polio, pertussis, measles, tetanus [8], influenza [9], and human papillomavirus (HPV) [10]. Perceived risks versus advantages, religious beliefs and a limited awareness were among the most common reasons cited [11,12]. Many studies have demonstrated that unhealthy behaviours influence vaccine acceptance, such as alcohol intake [13,14] and smoking habits [15,16]. There are mixed results regarding physical activity and vaccine uptake. Several studies have reported decreased physical activity as an obstacle to vaccination in some instances [17,18,19] and as a booster in other cases [13,20]. Thus, vaccine hesitancy has been studied through the lens of several cognitive and behavioural factors to date. Negative attitudes to vaccinations have been related to mistrust of authority segments of society, such as government officials, healthcare providers, and scientists [21,22,23,24]. Altogether, the evidence suggests that various psychological factors likely differentiate people who oppose vaccines and those who accept them.

The aforementioned determinants can also be adapted to the current COVID-19 vaccine hesitancy. Individuals who hesitate or refuse to vaccinate are characterized by more self-interest, distrust of specialists and authorities, greater adherence to religious beliefs, and the harbouring of conspiratorial and suspicious beliefs [25]. Moreover, people may use self-protection habits to replace vaccination in mitigating COVID-19. They may presume that conforming to such safety measures is sufficient for preventing infection [26]. This situation could be due to the spread of vaccine-related misinformation within society [27]. Furthermore, strong associations between intent to vaccinate and perceived safety [28], links between a negative attitude toward COVID-19 vaccines and the refusal to vaccinate [29], and the relationship between religiosity and a lesser degree of intent to vaccinate [30] highlight the need to understand the psychological factors contributing to vaccine hesitancy.

Further, many of the available works on vaccine hesitancy identify explicit reasons provided by people for opposing vaccination [9,31,32,33]. Although this knowledge is valuable, it is restricted in its capacity to elucidate why people arrive at their various epistemological positions [34]. Therefore, it may be more insightful to identify the psychological factors that characterize and differentiate individuals who hesitate to take or refuse vaccines from those who are responsive to vaccine programs. Thus, this systematic review aims to synthesise and integrate evidence on psychological factors of vaccine hesitancy in the pandemic context. Such a review can guide interventional programs designed to build and strengthen responses to combat the pandemic threat [35].

## 2. Methods

The current review was structured as per the updated guidelines for reporting systematic reviews [36].

### 2.1. Eligibility Criteria

The following inclusion criteria were used. The current review did not limit studies conducted solely among any specific group of participants as the study objective included understanding the psychological factors of vaccine hesitancy among different populations across the world. Studies were included if they investigated psychological factors associated with vaccine hesitancy. The search was limited to the English language. Further, articles were included if they were published from 2019 onward. The review chose this year as the cut-off as the analysis focused on the COVID-19 pandemic. The review excluded conference abstracts, unpublished manuscripts (preprints), commentaries, editorials, and publications that analysed only the secondary data.

### 2.2. Search Strategy

Online databases of PubMed, Scopus, Science Direct, PsycNET, and Web of Science were systematically examined using a combination of keywords: “cognitive”, “behavioural”, “determinant”, “emotional”, “psychological”, “vaccine hesitancy”, “vaccine refusal”, “vaccine opposition”, “vaccine reactance”, “vaccine resistance”, “vaccine acceptance”, “COVID-19”, and “SARS-CoV-2”. Boolean operators “AND” and “OR” were employed at this time to integrate keywords on each database. An additional literature search was conducted using Google Scholar to identify any other relevant articles.

### 2.3. Selection Process and Data Extraction

The first author (John Romate) completed the study conceptualization and came up with the search terms and carried out the search. The first three authors (John Romate., E.R. and A.G.) simultaneously screened the articles for the titles and abstracts independently. The identified references obtained through database search were exported to reference management software, Zotero, and then duplicates and retracted studies were removed. Next, the remaining citations were exported to a Microsoft Excel spreadsheet. These studies were screened against the eligibility criteria based on the titles and abstracts. Subsequently, a full-text review was conducted for articles with abstracts that met the eligibility criteria, again by the first three authors. The PRISMA flowchart was adhered to for each phase of article screening. After the full-text review of the studies for eligibility, data extraction was completed by the first two authors. The following data were extracted from each finalized article: author, year of publication, details concerning the country, sample information, and psychological factors.

### 2.4. Quality Assessment and Evidence Synthesis

The quality assessment of included studies was completed using critical appraisal tools from the Joanna Briggs Institute (JBI) [37]. These tools were scored on a rating scale of ‘yes’, ‘no’, ‘unclear’, and ‘not applicable’ across several study domains. Articles were appraised by the second and third authors (E.R. and A.G.) and a third reviewer decided on any discrepancies (John Romate). A narrative synthesis of extracted evidence was carried out comparing and contrasting the overall data and qualitatively presented as themes. The reviewers reached a consensus on the study findings through frequent discussions.

## 3. Results

### 3.1. Identification of Studies

**Figure 1:**
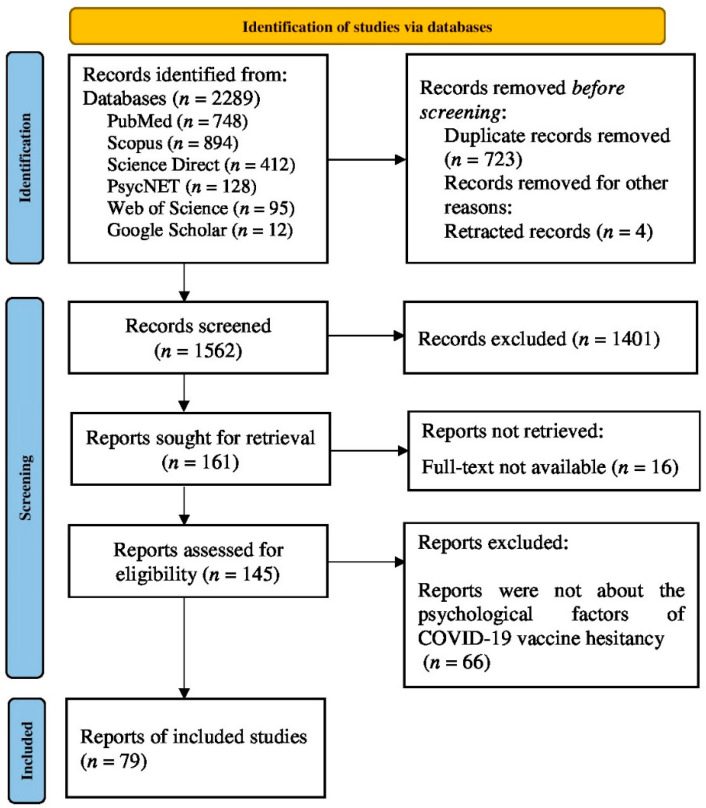
The PRISMA flow diagram depicting the selection of studies for the systematic review.

An initial search on five electronic databases yielded 2289 records, of which 748 were from PubMed, 894 from Scopus, 412 from Science Direct, 128 from PsycNET, and 95 from Web of Science. Further, an additional 12 studies were identified via the Google Scholar search. After deduplication and removal of retracted items, the remaining 1562 records were screened for selection based on the inclusion criteria (Figure 1). Subsequently, 1401 records were removed after the title and abstract screening. Of the 161 reports sought for retrieval, the full text was not available for 16 studies. The remaining 145 reports were assessed for eligibility. The full-text analysis excluded 66 reports that were not about the psychological factors of COVID-19 vaccine hesitancy. Thus, the final analysis included 79 quantitative studies on COVID-19 vaccination with an emphasis on the psychological factors associated with vaccine hesitancy.

### 3.2. Study Characteristics

Of the 79 studies selected for the final analysis, two were published in 2020, 58 were published in 2021, and the remaining 19 were published in 2022 (Table 1). The included studies were conducted in the United States (US) *(n* = 10), China (*n* = 5), UK (*n* = 4), Saudi Arabia (*n* = 4), Italy (*n* = 3), Kuwait (*n* = 3), India (*n* = 3), Bangladesh (*n* = 3), South Korea (*n* = 3), Jordan (*n* = 3), Turkey (*n* = 2), Tunisia (*n* = 2), Qatar (*n* = 2), Turkey (*n* = 2), Thailand (*n* = 2), Ireland & UK (*n* = 2), Hong Kong (*n* = 2), and one study each from Malta, Austria, Canada, Pakistan, Palestine, France, Egypt, Iran, Mexico, Mongolia, Norway, Brazil, UAE, Africa, Ethiopia, Cyprus, Greece, Portugal, Australia, Iraq, Zimbabwe, and Nigeria. Of the remaining two studies, one was conducted across nine low- and middle-income countries and the other was in Jordan, Kuwait, and other Arab countries. The selected studies included those completed prior to COVID-19 vaccine authorization (which analysed the psychological factors of future vaccine hesitancy by assuming that vaccines would be available) and those studies conducted after the authorization of COVID-19 vaccines. All the finalized articles used cross-sectional designs (*n* = 79). Most of the studies were conducted among the general population (*n* = 48). Other studies covered healthcare workers, medical students, university students, parents, physicians, mothers with a mental health history, vaccine priority population, adults with multiple sclerosis, nursing students, nurses and midwives, college students, and pregnant and lactating women.

### 3.3. Quality Assessment

The quality assessment of 79 studies included in the current systematic review was conducted using JBI critical appraisal tools. The risk of bias for the assessed studies was generally at a moderate to high level. Moreover, no studies were eliminated based on the level of quality appraisal. The quality assessment results can be found in the supplementary file.

### 3.4. Psychological Factors Associated with Vaccine Hesitancy

The current review findings provide a comprehensive list of various psychological factors associated with vaccine hesitancy but further suggest such factors could be conceptualized into seven main themes: appraisals of the COVID-19 pandemic, vaccine safety and side effects, general vaccine confidence/trust, trust in government and healthcare professionals, scepticism around vaccine production, conspiracy beliefs, emotions, and information and knowledge about the vaccine (Table 2).

#### 3.4.1. Appraisal of COVID-19 Pandemic

The literature review clearly evidences the association between appraisals of COVID-19 and vaccine hesitancy. Specifically, vaccine hesitancy was reported more likely among respondents with little to no fear of COVID-19 infection [42,45,46,51,62,69,75]. One study revealed that respondents who considered the vaccination to be unnecessary and with lower perceived danger of COVID-19 with greater vaccine hesitancy showed vaccine complacency [79]. Further, individuals who experienced no symptoms during the pandemic were more likely to report vaccine hesitancy [83]. Thus, participants who more strongly perceived their risk of being infected by COVID-19 as lower demonstrated a higher tendency toward vaccine hesitancy [84]. Similarly, vaccination was accepted by more people who were afraid of COVID-19 than those who were not [105]. Specifically, a study including an Irish and UK sample reported higher fear of COVID-19 among the vaccine accepting groups than those who were vaccine-hesitant [115].

#### 3.4.2. Vaccine Safety and Side Effects

One theme extracted from the investigated studies was that perceptions of the safety and side effects of the COVID-19 vaccine had a greater influence on vaccine hesitancy. Participants’ concerns regarding the safety and efficacy of the COVID-19 vaccine were found in many studies [38,39,41,44,48,49,50,53,57,58,63,71,76,77,78,83,84,85,86,88,89,96,102,104,111,115]. More evidently, the respondents in a reviewed study reported 29 reasons for vaccine hesitancy/rejection, wherein the top reason was safety concerns about vaccines [59]. Moreover, people perceived vaccines as unsafe [94] and believed that vaccines may interfere with the treatment outcome or efficacy of other medical/health conditions [75,81]. Whereas, some individuals were hesitant to uptake the vaccine because of the possible side effects of vaccines, as reported in Refs. [48,53,56,57,58,59,60,63,65,71,75,80,86,88,89,92,93,95,96,97,98,100,101,104,107,114]. Concerns about side effects and the efficacy of the vaccine were perceived as barriers that negatively influence willingness to accept vaccination [39]. The findings revealed that such concerns may range from possible vaccine side effects, beliefs regarding the disease itself, people’s perception of rushing to conduct vaccine trials, profiteering of pharmaceutical companies from vaccines, and preferred dependence on natural immunity. In general, participants who were ready to receive a vaccine against COVID-19 showed lesser concerns when compared to individuals who are hesitant to vaccinate.

#### 3.4.3. Vaccine Confidence/Trust

Individual vaccine confidence/trust in general was found to negatively correlate with COVID-19 vaccine hesitancy. The findings emphasized that respondents with high levels of vaccine confidence or trust in general reported low vaccine hesitancy when compared with those people who had low vaccine trust [42,44]. Several studies reported the association of low confidence in vaccinating against COVID-19 or vaccines in general with vaccine hesitancy [61,65,83,103,105]. Further, mistrust in the vaccine made many individuals unwilling to get vaccinated [42,53,56,98]. Moreover, individuals who were less likely to have received previous vaccines against influenza were less likely to receive a COVID-19 vaccine [38,45]. Previous vaccination behaviour against the flu increased the intention to uptake the vaccine but decreased with an increase in general doubts regarding the vaccine [40]. In one study, participants reported uncertainty and mistrust in vaccines as the most common reason for avoiding COVID-19 vaccination [60].

#### 3.4.4. Trust in Government and Healthcare Professionals

The findings identified medical mistrust as a major cognitive factor influencing vaccine hesitancy during the COVID-19 pandemic. Some of the studies revealed that, during the COVID-19 pandemic, there was widespread medical distrust that made a vast number of people refuse vaccination [25,59,69,72,75,82,115]. Moreover, lack of trust in the government led to vaccine hesitancy by generating concerns about the vaccination information provided by government agencies [25,47,50,62,69,77,84,98,104,108,115]. Specifically, in one study, slightly more than half of the participants lacked trust in the ability of governments and other relevant authorities in ensuring the availability of a safe and effective vaccine [111]. Another study reported that trust in the government or voting behaviour was related to vaccine hesitancy. People who voted for opposition parties or did not even vote were more likely to hesitate than respondents who voted for the governing parties [47]. Further, “anti-vaccine” attitudes were also found to be related with “anti-authority” attitudes [25,46].

#### 3.4.5. Scepticism around Vaccine Production

Expedited vaccine production is reported as a contributing factor to vaccine hesitancy across many studies [59,71,95,100,109]. The individual assumption that vaccines were developed rapidly without reasonable trial duration and with safety issues may result in hesitancy to accept their vaccination to ensure effectiveness [41]. Relatedly, mistrust in vaccine-developing companies [59], pharmaceutical lobbying [82], and policymakers’ and managers’ motivations to recommend the vaccine [83] were also reported as concerns that led people to refuse or delay COVID-19 vaccination. Moreover, less trust in science or scientists [25,61,62] has influenced perceptions of people about vaccination.

#### 3.4.6. Conspiracy Beliefs

The evidence suggested that people who reported vaccine hesitancy were less likely to receive pandemic-related information from sources including healthcare professionals and scientists [46], and their perception of the causes of COVID-19 largely constituted conspiracy theories held by individuals [25,46,54,62,66,67,68,69,72,110]. For instance, participants in one study had a conspiracy belief that COVID-19 has an “artificial origin” [46], whereas another study reported individuals’ belief in a pre-planned pandemic [99]. Further, participants in another study revealed conspiracy beliefs such as the injection of microchips into recipients and infertility related to vaccination, respectively [68]. Relatedly, the findings revealed that people who exhibited vaccine hesitancy reported that they were concerned about misinformation related to the vaccine [54,64,67]. Whereas, addressing misinformation on the COVID-19 vaccine can enhance public confidence in healthcare experts, mitigate the effects of conspiracy beliefs, and motivate individuals to follow COVID-19 preventive measures [69].

#### 3.4.7. Emotions

People’s anxiety about COVID-19 vaccines and their rapid production can result in vaccine hesitancy [46,59,71,95,100,109]. Relatedly, worry that the COVID-19 vaccine might adversely affect their present medical/health condition may make people unlikely to obtain the COVID-19 vaccine [75,81,109]. The findings also revealed that people with less fear of COVID-19 were more likely to exhibit vaccine hesitation [42,45,46,115]. Conversely, the findings from another study suggested that individuals who refused to vaccinate had low levels of anxiety, were less worried about the current pandemic, and found the pandemic to be media hype that induced fear. Moreover, their level of resilience perception was high [69]. Further, individuals who reported fear of injection were more likely to hesitate to accept COVID-19 vaccination than individuals who reported no such fear [43,44,105]. Conversely, concerns of losing loved ones to COVID-19 and worries regarding healthcare system overload were found as positive predictors of willingness to uptake the vaccine [69].

#### 3.4.8. Information and Knowledge about Vaccines

The findings indicated social media platforms as a major source of information on COVID-19 vaccines [57,62,66,68,70,73,76]. Further, individuals who were resistant to vaccination expressed less reliance and trust in authoritative and traditional sources of information [25] and broadcast and print media information [62]. Meanwhile, participants in one study indicated healthcare and social service providers as the most trusted sources of vaccination-related information [62]. Conversely, findings from another study indicated that individuals who reported vaccine hesitancy were less likely to receive pandemic-related information from sources including healthcare professionals and scientists [46]. Moreover, inconsistent information from elected authorities and public health professionals was found to influence vaccine hesitancy [103]. In addition, a lack of correct information on the COVID-19 vaccines acts as a potential barrier to COVID-19 vaccine uptake [38,44,58,60,102]. Besides, individuals who were unaware of the vaccine type authorized in their nations were more likely to exhibit vaccine hesitancy [59]. Furthermore, another study revealed that low levels of knowledge of the preventive measures related to COVID-19 led to vaccine refusal [69].

## 4. Discussion

Vaccine hesitancy acts as a potential threat to global health and limits the health system’s ability to contain the spread of the virus. The aim of the current systematic review was to integrate available evidence on the psychological factors contributing to vaccine hesitancy. The findings reveal an association of increased risk perception with greater vaccine hesitancy. These findings during the pandemic are consistent with previous studies that have revealed risk perception as a robust predictor of protective health behaviours and prevention intention, which includes vaccine uptake [116]. The findings further indicate that the safety and possible side effects of the COVID-19 vaccine play a crucial role in vaccine hesitancy. Research before the pandemic showed that concerns about safety and side effects of vaccines are among the essential factors influencing decisions to vaccinate, specifically for newly produced vaccines [32,117,118]. Similarly, uncertainty and mistrust in vaccines were the most common reason to avoid vaccination. Individuals with more doubts regarding vaccines in general were less willing to receive vaccination. Moreover, the current review findings are in line with prior studies that reported that those who received vaccination against seasonal flu in 2019 were more likely to vaccinate against new pandemic diseases [119,120]. Although vaccine hesitancy has been characterized as vaccine-specific and context-specific [2], the current review suggests that it is plausible that, the more individuals who had concerns about vaccinations in general, the less likely they were to uptake any type of vaccine [40]. Thus, it is critical to provide information regarding the efficacy, safety, and side effects of COVID-19 vaccines to people [121] considering that the reviewed studies highlight the crucial role of such information in combating vaccine hesitancy [57,58].

The findings further reveal that individuals’ trust in health professionals is associated with their intention to vaccinate. This result was consistent with a study conducted before the pandemic indicating that physician recommendation is strongly correlated with vaccine acceptability among patients [122]. Meanwhile, medical mistrust, which is described as an absence of trust in healthcare professionals, the healthcare system, medical treatments, and the government as a custodian of public health [123,124], is identified as a major cognitive factor influencing vaccine hesitancy during the COVID-19 pandemic. Moreover, rapid development of COVID-19 vaccines resulted in a low willingness to vaccinate. A recent review reveals that mass production of vaccines, equitable distribution of those vaccines over the world, and uncertainty about their long-term efficacy are the main obstacles that could prevent COVID-19 vaccination programmes from being successfully implemented [125]. The findings further show that a lack of trust in vaccine manufacturers, governments, and health care providers can lead to the backing of conspiracy beliefs that can cause a negative impact on public health due to their contribution to vaccine hesitancy. The acceptance of such beliefs could be connected to concerns about the vaccine’s perceived safety and the uncertainty about COVID-19 vaccine benefits; a similar trend was observed in studies on influenza vaccine hesitancy [117,126]. Similarly, individuals susceptible to conspiracy beliefs may ignore the interventions developed by scientists and medical professionals [127]. Thus, the review emphasizes that the healthcare professionals should update the public on vaccine-related information, both verified as well as uncertain information, which, in turn, helps to develop trust in healthcare professionals and authorities.

An earlier study before the current pandemic linked negative emotions with vaccine attitudes and vaccination risk perceptions [128]. The findings on emotions and vaccine hesitancy during the current COVID-19 pandemic also confirmed this trend. Establishing a balance between the pandemic perceptions of individuals and their emotional response to the pandemic was viewed as important as these factors were found to affect vaccination behaviour. The current review identifies the need for including emotionally compelling ideas in vaccine promotion, along with strengthening the credibility and trust in government authorities and experts.

Further, the degree of individual knowledge and information regarding the illness and vaccine are crucial in achieving herd immunity as they influence vaccine uptake. The findings show an increased focus on media platforms, particularly social media, in shaping individual opinion on the COVID-19 pandemic and vaccinations. However, uncritical usage of social media information was more likely to increase vaccine hesitancy. Instant access and wide communication between users when coupled with anonymity provided an immense ability for social media to propagate unvetted and unverified information. For instance, people who exhibited vaccine hesitancy report the need to address misinformation related to the safety of the vaccine [45]. The World Health Organization has also raised an alert on the need to combat the “infodemic”, another form of epidemic that quickly spreads misleading information, fake news, and incorrect scientific claims [129]. Moreover, social media algorithms allowed audiences to follow content that conformed to their views and rejected contrasting views, leading to the formation of communities who subscribe to particular ideologies and opinions [130]. Past outbreaks of SARS, Ebola, and H1N1 have highlighted the pivotal role of health-related information in vaccine acceptance and disease prevention [131]. Thus, the findings stress the importance of credible and reliable information on COVID-19 vaccines to reduce vaccine hesitancy and eradicate misinformation on social media.

## 5. Implications, Limitations, and Future Recommendations

While the world expects COVID-19 vaccines to protect public health and prevent the collapse of healthcare systems, the current review reveals vaccine hesitancy (and consequent vaccine refusal) as a potential barrier. On the other hand, the findings suggest that psychological factors underlying vaccine hesitancy can be effectively used to design future vaccination campaigns that can deal with vaccine hesitancy. Further, understanding the psychological determinants can provide a suitable direction and knowledge for intervention developments. As the COVID-19 pandemic continues with new variants, achieving herd immunity is the ultimate goal, and, in this context, the findings of the current review can be extremely beneficial toward increasing vaccine acceptance and to prepare for any similar future crises.

The current study concentrated on psychological factors influencing vaccination hesitancy. However, there may be varying degrees of connection between psychological factors and certain vaccinations. However, vaccine-type-based findings were not reported in the current review. Additionally, it is possible that significant distinctions may exist between hesitancy, refusal, and opposition, all of which require future study. Because the studies reviewed were cross-sectional, causal conclusions between psychological factors and vaccination hesitancy require future approaches with greater care. More longitudinal or intervention studies are thus required. Further, the review might have classified each psychological factor by country when identifying the contributing factors to vaccine hesitancy, which would have made it simpler to comprehend the underlying reasons for COVID-19 vaccine hesitancy in each nation. Moreover, the psychological characteristics of vaccine-hesitancy may change over time due to the increasing availability of scientific data on COVID-19 vaccinations. Thus, further studies may be needed to identify and analyse these changes over time. However, the review attempted to provide a comprehensive understanding of the psychological factors of vaccine hesitancy by including articles from 2020 to 2022.

The adverse effect of vaccine hesitancy on the development and implementation of mass vaccination programmes needs to be managed with evidence-based vaccine information and effective and proactive measures to fight misinformation. It is important to assign expert groups of scientists and healthcare professionals to provide accurate and reliable data on vaccination in order to reduce vaccine ambiguity and distrust among the public. Healthcare practitioners need to listen to the public concerns, answer their questions, and counter misinformation. Moreover, social media need to pay considerable attention to misleading information regarding vaccination. Besides, it is crucial to conduct studies on vaccine hesitancy by considering conspiracy theories as the general beliefs of people regarding conspiracy theories can be reflected in their vaccine-related attitudes.

## 6. Conclusions

Vaccine hesitancy is a major challenge to public health during pandemics. This systematic review focused on the psychological factors of vaccine hesitancy and reported the crucial determinants found to be common across countries and different demographic groups. The most common reason for vaccine hesitancy was its safety and side effects. However, conspiracy beliefs and using social media platforms to spread vaccine-related misinformation have also challenged the acceptance of vaccines worldwide. The lack of adequate vaccine information highlights the need to disseminate high-quality and reliable information to enhance vaccine acceptance and coverage. Extensive vaccination campaigns and educational initiatives are required in concert with vaccination promotion efforts to address the psychological factors contributing to vaccine hesitancy. Thus, the government and healthcare professionals need to focus on various cognitive, behavioural, and emotional characteristics of people to successfully cope with vaccine hesitancy and achieve herd immunity.

## Figures and Tables

**Table 1:** Summary of study characteristics.

Sl. No.	Author(s) & Year	Country	Sample	Sample Size	Associated Psychological Factors
1	Murphy et al. [25]	Ireland & UK	General population	*n* = 3066	Mistrust in authoritative and traditional information sources of pandemic, less trust in healthcare professionals, state and scientists, lower cognitive reflection, high social dominance and authoritarianism, negative attitudes toward migrants, lower levels of altruism, high conspiracy and religious beliefs, low personality trait agreeableness, high internal locus of control
2	Fisher et al. [38]	United States	General population	*n* = 991	Not received the influenza vaccine in the previous year, vaccine-specific concerns, inadequate information, anti-vaccine attitudes or beliefs, lack of trust
3	Lin et al. [39]	China	General population	*n* = 3541	Concerns about vaccine side effects and efficacy
4	Caserotti. [40]	Italy	General population	*n* = 2267	Doubts about the vaccines in general
5	Alqudeimat et al. [41]	Kuwait	General population	*n* = 2368	Vaccine’s health-related risks and concerns
6	Willis et al. [42]	United States	General population	*n* = 1205	No fear of COVID-19 infection, low vaccine trust in general
7	Freeman et al. [43]	UK	General population	*n* = 15,014	Injection fear
8	Cordina et al. [44]	Malta	General population	*n* = 3363	Lack of vaccine safety, fear of injections, need more information about the vaccine
9	Yang et al. [45]	United States	Adults with a history of tobacco or marijuana use	*n* = 387	Not stressed because of the COVID-19, previous influenza vaccination behaviour
10	Nazli et al. [46]	Turkey	General population	*n* = 467	Belief in conspiracy theories, low fear of COVID-19
11	Schernhammer et al. [47]	Austria	General population	*n* = 1007	Voting behaviour or trust in the government
12	Altulahi et al. [48]	Saudi Arabia	General population	*n* = 8056	Vaccine side effects and safety
13	Aloweidi et al. [49]	Jordan	Medical and non-medical workers	*n* = 646	Rumour that vaccines are not safe
14	Benham et al. [50]	Canada	General population	*n* = 4498	Vaccine side effects, low influence by peers or health care professionals, low trust in government institutions
15	Chaudhary et al. [51]	Pakistan	General population	*n* = 423	Lack of knowledge, understanding, and perception of the risk, safety
16	Chen et al. [52]	China	General population	*n* = 2531	Perception of COVID-19 susceptibility, perceived barriers to vaccination
17	Danabal et al. [53]	India	General population	*n* = 564	Adverse effects, mistrust in vaccines
18	Hossain et al. [54]	Bangladesh	General population	*n* = 1497	Conspiracy beliefs, widespread misinformation, superstitions about the COVID-19 vaccine
19	Hossain et al. [55]	Bangladesh	University students	*n* = 900	Inadequate knowledge, negative vaccine perceptions and attitudes
20	İkiışık et al. [56]	Turkey	General population	*n* = 384	COVID-19 risk perception
21	Alabdulla et al. [57]	Qatar	Migrant majority population	*n* = 7821	Concerns around the COVID-19 vaccine safety and its longer-term side effects
22	Saied et al. [58]	Egypt	Medical students	*n* = 2133	Concerns about the vaccine’s ineffectiveness and adverse effects, insufficient data on the adverse effects of vaccine, inadequate information regarding the vaccine.
23	Qunaibi et al. [59]	Jordan	General population	*n* = 36,220	Concerns about vaccine side effects, expedited vaccine production, distrust in health care policies, vaccine-developing companies, and published studies, deficient data regarding vaccine type authorized in their countries
24	Faezi et al. [60]	Iran	General population	*n* = 1880	Fear of vaccination-related illness, concern about vaccine side effects, lack of reliable information about vaccine promotion
25	Milan & Dau [61]	United States	Mothers with a mental health history	*n* = 240	Low confidence in vaccinating against COVID-19, less belief in science, less influence from healthcare and governmental sources
26	Allington et al. [62]	UK	General population	*n* = 4343	High reliance on social media information, less reliance on broadcast and print media information, reduced COVID-19 perceived risk, decreased trust in medics, scientists, and in government, coronavirus conspiracy suspicions
27	Xu et al. [63]	China	Parents	*n* = 4748	Concerns about COVID-19 vaccine effectiveness and side effects
28	Castaneda-Vasquez [64]	Mexico	Health professionals	*n* = 543	Misinformation related to vaccination and COVID-19
29	Bono et al. [65]	Nine Low- and Middle-Income Countries	General population	*n* = 10,183	Less confidence in vaccine effectiveness, fear of vaccine side effects
30	Al-Sanaf & Sallam [66]	Kuwait	Healthcare workers	*n* = 1019	Vaccine conspiracy beliefs, sources of knowledge about COVID-19 vaccines, such as social media platforms
31	Sallam et al. [67]	Jordan, Kuwait and other Arab countries	General population	*n* = 3414	Conspiracy beliefs, COVID-19 misinformation
32	Sallam et al. [68]	Jordan	University students	*n* = 1106	Conspiracy beliefs, dependence on social media platforms
33	Kuçukkarapinar et al. [69]	Turkey	General population	*n* = 3888	Conspiracy theories, lesser compliance with preventive measures, less knowledge about prevention, decreased risk perception, increased media hype, reduced trust in government and medical professionals
34	Plitch-loeb et al. [70]	United States	Vaccine priority population	*n* = 2650	Vaccine information from social media or both social media and traditional channels
35	Alibrahim & Awad [71]	Kuwait	General population	*n* = 4147	Possible side effects of the vaccine, quick development, efficacy in infection prevention, negative attitude regarding vaccines in general
36	Acar-Burkay & Cristian [72]	UK	General population	*n* = 435	COVID-19 conspiracy beliefs, trust in healthcare authorities
37	Dambadarjaa et al. [73]	Mongolia	General population	*n* = 2875	Social media reliance, COVID-19 vaccine type and side effects
38	Ebrahimi et al. [74]	Norway	General population	*n* = 4571	Perceived risk of COVID-19 vaccines, belief in the power of natural immunity, preference to unmonitored media platforms
39	Ehde et al. [75]	United States	Adults with multiple sclerosis	*n* = 359	Lower risk perception of COVID-19, lower trust in healthcare officials, concerns about the vaccine’s long-term effects, vaccine’s impact on health history/conditions
40	Almaghaslah et al. [76]	Saudi Arabia	General population	*n* = 862	Vaccine effectiveness, news on social media
41	Jain et al. [77]	India	Medical students	*n* = 1068	Vaccine efficacy and safety, lack of trust in government agencies, limited awareness about vaccination eligibility
42	Kumar et al. [78]	Qatar	Healthcare workers	*n* = 7821	Safety and efficacy concerns of vaccine
43	Luk et al. [79]	Hong Kong	General population	*n* = 1501	Insufficient knowledge about COVID-19 transmission, low COVID-19 perceived danger
44	Maraqa et al. [80]	Palestine	Healthcare workers	*n* = 1159	Vaccine’s side effects
45	Mejri et al. [81]	Tunisia	Cancer patients	*n* = 329	Vaccine’s interference with treatment efficacy or treatment outcome
46	Navarre et al. [82]	France	Hospital workers	*n* = 1964	Distrust in health authorities and pharmaceutical lobbying
47	Oliveira et al. [83]	Brazil	General population	*n* = 4630	Low confidence in vaccine safety and efficacy, in the healthcare system, or in policymakers’ and managers’ motivations to recommend vaccine, low immune preventable diseases risk perception, considering vaccination unnecessary
48	Park et al. [84]	South Korea	General population	*n* = 1000	COVID-19 risk perceptions, vaccine safety, self-rated government trust, and political ideologies
49	Sethi et al. [85]	UK	General population	*n* = 4884	Vaccine’s possible side effects
50	Sirikalyanpaiboon et al. [86]	Thailand	Physicians	*n* = 705	Uncertainty of the vaccine efficacy, fear of adverse events
51	Yahia et al. [87]	Saudi Arabia	General population	*n* = 531	Belief that vaccines are futile or hazardous
52	Yeşiltepe et al. [88]	Turkey	Nursing students	*n* = 1167	Concerns regarding vaccine’s side effects, limited evidence on effectiveness and reliability
53	Albahri et al. [89]	UAE	General population	*n* = 2705	Vaccine side effects and safety, belief that one needs to develop natural immunity
54	Singh et al. [90]	Hong Kong	General population	*n* = 245	Negative attitudes towards COVID-19 vaccine
55	Ali & Hossain [91]	Bangladesh	General population	*n* = 1134	Doubtful of the vaccine’s efficacy
56	Anjorin et al. [92]	Africa	General population	*n* = 5416	Serious side effects of vaccine
57	Boon-Itt et al. [93]	Thailand	General population	*n* = 862	Potential harmful side effects of a COVID-19 vaccine
58	Yilma et al. [94]	Ethiopia	Healthcare workers	*n* = 1314	Perception that vaccines are unsafe
59	Fakonti et al. [95]	Cyprus	Nurses and Midwives	*n* = 437	Expedited development of vaccines and fear of side effects
60	Li et al. [96]	China	Medical students	*n* = 2196	Fear of vaccine’s consequences, concerns about short-term side effects and ineffectiveness
61	Magadmi et al. [97]	Saudi Arabia	General population	*n* = 3101	Concerns about side effects
62	Khairat et al. [98]	United states	General population	*n* = 3142	Lack of vaccine trust, concerns regarding vaccine side effects, lack of trust in government
63	Holeva et al. [99]	Greece	General population	*n* = 538	Belief in a pre-planned pandemic
64	Hubach et al. [100]	United states	General population	*n* = 222	Limited understanding and knowledge regarding the vaccine, including long-term complications, potential side effects, and scepticism around COVID-19 vaccine efficacy and development
65	Lo Moro et al. [101]	Italy	Medical students	*n* = 929	Adverse reactions after a vaccination, relative’s advice against COVID-19 vaccination
66	Silva et al. [102]	United states	College students	*n* = 237	Concerns about vaccine effectiveness and safety, limited information
67	Soares et al. [103]	Portugal	General population	*n* = 1943	Reduced confidence in COVID-19 vaccine and the healthcare service, perception of the information provided as contradictory and inconsistent, worse perception of government actions
68	Kavanagh et al. [104]	Australia	Disability support workers	*n* = 252	Inadequate safety data, side effects, distrust in the government
69	Hwang et al. [105]	South Korea	General population	*n* = 13,012	Lack of COVID-19 vaccine confidence, less or no COVID-19 fear
70	Hong et al. [106]	China	Cancer patients	*n* = 2158	Worry that the COVID-19 vaccine might worsen the prognosis of cancer
71	Shareef et al. [107]	Iraq	General population	*n* = 1221	Concerns about vaccine’s future side effects
72	Lee & You [108]	South Korea	General population	*n* = 1016	Perceived barriers of vaccination, lower trust in government
73	Kumari et al. [109]	India	Pregnant and lactating women	*n* = 313	Concerns about the vaccine’s future effects on the foetus, rushed development
74	Moscardino et al. [110]	Italy	General population	*n* = 1177	Conspiracy theories and negative attitudes toward vaccines
75	Mundagowa et al. [111]	Zimbabwe	General population	*n* = 1168	Uncertainty about the safety and effectiveness of the vaccine, lack of trust in the government’s ability to ensure effective vaccine availability
76	Zammit et al. [112]	Tunisia	Health professionals	*n* = 493	Concerns regarding components of vaccines
77	Ekowo et al. [113]	Nigeria	General population	*n* = 1283	Belief in one’s own immunity, side effects of the vaccine
78	Skeens et al. [114]	United states	Parents of children with cancer	*n* = 491	Concerns regarding vaccine side effects on children
79	Walsh et al. [115]	Ireland & UK	General population	*n* = 1079	Low peer influence, lower satisfaction with government response, low fear of COVID-19, low civic responsibility, low adherence to healthcare guidelines, low trust in authorities, low positive vaccination attitudes, perceived risk of COVID-19 vaccine, low perceived vaccine benefit, perceived vaccine severity, low perceived susceptibility

**Table 2:** Overview of psychological factors related to vaccine hesitancy.

Author(s)	No. of Studies	Major Themes	Sub-Themes
Willis et al. [42]; Yang et al. [45]; Nazli et al. [46]; Chaudhary et al. [51]; Chen et al. [52]; İkiışık et al. [56]; Allington et al. [62]; Kuçukkarapinar et al. [69]; Ehde et al. [75]; Luk et al. [79]; Oliveira et al. [83]; Park et al. [84]; Hwang et al. [105]; Walsh et al. [115]	14	Appraisal of COVID-19	Low perceived susceptibility to virus Low perceived severity of disease No fear of COVID-19
Fisher et al. [38]; Lin et al. [39]; Alqudeimat et al. [41]; Cordina et al. [44]; Altulahi et al. [48]; Aloweidi et al. [49]; Benham et al. [50]; Chaudhary et al. [51]; Danabal et al. [53]; Alabdulla et al. [57]; Saied et al. [58]; Qunaibi et al. [59]; Faezi et al. [60]; Xu et al. [63]; Bono et al. [65]; Alibrahim & Awad [71]; Dambadarjaa et al. [73]; Ebrahimi et al. [74]; Ehde et al. [75]; Almaghaslah et al. [76]; Jain et al. [77]; Kumar et al. [78]; Maraqa et al. [80]; Mejri et al. [81]; Park et al. [84]; Sethi et al. [85]; Sirikalyanpaiboon et al. [86]; Yahia et al. [87]; Yeşiltepe et al. [88]; Albahri et al. [89]; Ali & Hossain [91]; Anjorin et al. [92]; Boon-Itt et al. [93]; Yilma et al. [94]; Fakonti et al. [95]; Li et al. [96]; Magadmi et al. [97]; Khairat et al. [98]; Hubach et al. [100]; Lo Moro et al. [101]; Silva et al. [102]; Kavanagh et al. [104]; Shareef et al. [107]; Lee & You [108]; Kumari et al. [109]; Mundagowa et al. [111]; Ekowo et al. [113]; Skeens et al. [114]; Walsh et al. [115]	49	Vaccine safety and side effects	Vaccine is unsafe Vaccines are dangerous Concern about vaccination Vaccine causes side effects Vaccine’s health-related concerns Concerns about components of vaccines
Fisher et al. [38]; Caserotti. [40]; Willis et al. [42]; Danabal et al. [53]; Hossain et al. [55]; İkiışık et al. [56]; Milan & Dau [61]; Alibrahim & Awad [71]; Ebrahimi et al. [74]; Oliveira et al. [83]; Yahia et al. [87]; Albahri et al. [89]; Singh et al. [90]; Ali & Hossain [91]; Khairat et al. [98]; Soares et al. [103]; Hwang et al. [105]; Moscardino et al. [110]; Ekowo et al. [113]; Walsh et al. [115]	20	General vaccine confidence/trust	Disagree with immunization Vaccination is unnecessary No confidence in value of vaccines Anti-vaccine attitudes or beliefs Low vaccine trust in general Belief in the power of natural immunity
Murphy et al. [25]; Fisher et al. [38]; Schernhammer et al. [47]; Benham et al. [50]; Qunaibi et al. [59]; Milan & Dau [61]; Allington et al. [62]; Kuçukkarapinar et al. [69]; Acar-Burkay & Cristian [72]; Ehde et al. [75]; Jain et al. [77]; Navarre et al. [82]; Oliveira et al. [83]; Park et al. [84]; Khairat et al. [98]; Soares et al. [103]; Kavanagh et al. [104]; Lee & You [108]; Mundagowa et al. [111]; Walsh et al. [115]	20	Trust in the healthcare professionals and government	No trust in the government Perceived government pressure to vaccinate Low influence of healthcare provider
Murphy et al. [25]; Qunaibi et al. [59]; Milan & Dau [61]; Allington et al. [62]; Alibrahim & Awad [71]; Navarre et al. [82]; Oliveira et al. [83]; Fakonti et al. [95]; Hubach et al. [100]; Kumari et al. [109]	10	Scepticism around vaccine production	Expedited vaccine production Distrust in vaccine-developing companies Lack of trust in scientists Less belief in science Pharmaceutical lobbying
Murphy et al. [25]; Nazli et al. [46]; Aloweidi et al. [49]; Hossain et al. [54]; Allington et al. [62]; Castaneda-Vasquez [64]; Al-Sanaf & Sallam [66]; Sallam et al. [67]; Sallam et al. [68]; Kuçukkarapinar et al. [69]; Acar-Burkay & Cristian [72]; Holeva et al. [99]; Moscardino et al. [110]	13	Conspiracy beliefs	Origin of vaccine Biological weapon Media hype Misinformation/disinformation Belief in conspiracy theories
Freeman et al. [43]; Cordina et al. [44]; Yang et al. [45]; Nazli et al. [46]; Faezi et al. [60]; Xu et al. [63]; Sirikalyanpaiboon et al. [86]	7	Emotions	Worry about vaccine Injection fear No stress because of COVID-19 Fear of vaccine-related illness Fear of adverse events
Murphy et al. [25]; Fisher et al. [38]; Cordina et al. [44]; Chaudhary et al. [51]; Hossain et al. [55]; Saied et al. [58]; Qunaibi et al. [59]; Faezi et al. [60]; Allington et al. [62]; Al-Sanaf & Sallam [66]; Sallam et al. [68]; Kuçukkarapinar et al. [69]; Plitch-loeb et al. [70]; Dambadarjaa et al. [73]; Ebrahimi et al. [74]; Almaghaslah et al. [76]; Jain et al. [77]; Luk et al. [79]; Yeşiltepe et al. [88]; Hubach et al. [100]; Silva et al. [102]; Soares et al. [103]; Kavanagh et al. [104]	23	Information and knowledge about vaccine	Inadequate knowledge about vaccine Incorrect knowledge Lack of scientific data Less satisfaction with information Influence of information through social media Perceived lack of information for vaccination decision

## Data Availability

All data relevant to the study are included in the article or uploaded as supplementary information.

## Supplementary Materials

The following supporting information can be downloaded at: https://www.mdpi.com/article/10.3390/vaccines10111777/s1. Supplementary File S1: PRISMA 2020 checklist; Supplentary File S2: Quality assessment results.
