# Supplementary material for: What Contributes to COVID-19 Vaccine Hesitancy? A Systematic Review of the Psychological Factors Associated with COVID-19 Vaccine Hesitancy
Source: Vaccines (Basel). 2022 Oct 22;10(11):1777. doi: 10.3390/vaccines10111777 (PMC9698528; doi:10.3390/vaccines10111777)
Supplement: Supplementary file 1 [file vaccines-10-01777-s001.zip › S2- Supplentary file-Quality assessment results.pdf]

Supplement 1. Quality assessment results of cross-sectional studies using Joanna Briggs Institute (JBI) Checklist

Sl. No.	Author(s) & Year	Q1[a]	Q2	Q3	Q4	Q5	Q6	Q7	Q8	% Yes	Risk[b]
1	Murphy et al. [25]	Y	Y	Y	Y	N	N	Y	Y	75	Low
2	Fisher et al. [38]	Y	Y	Y	Y	U	U	Y	Y	75	Low
3	Lin et al. [39]	Y	Y	Y	Y	U	U	Y	Y	75	Low
4	Caserotti. [40]	Y	Y	Y	Y	Y	N	Y	Y	87.5	Low
5	Alqudeimat et al. [41]	Y	Y	Y	Y	N	N	Y	Y	75	Low
6	Willis et al. [42]	Y	Y	N	Y	N	N	N	Y	50	Moderate
7	Freeman et al. [43]	Y	Y	Y	Y	Y	Y	Y	Y	100	Low
8	Cordina et al. [44]	Y	Y	N	Y	N	N	Y	Y	62.5	Moderate
9	Yang et al. [45]	Y	Y	N	N	N	N	Y	Y	50	Moderate
10	Nazli et al. [46]	Y	Y	Y	Y	N	N	Y	Y	75	Low
11	Schernhammer et al. [47]	Y	Y	Y	Y	N	N	Y	Y	75	Low
12	Altulahi et al. [48]	Y	Y	Y	U	N	NA	Y	Y	62.5	Moderate
13	Aloweidi et al. [49]	Y	Y	Y	Y	N	NA	Y	Y	75	Low
14	Benham et al. [50]	Y	Y	Y	Y	N	NA	Y	Y	75	Low

Supplement 1 *Cont.*

Sl. No.	Author(s) & Year	Q1[a]	Q2	Q3	Q4	Q5	Q6	Q7	Q8	% Yes	Risk[b]
15	Chaudhary et al. [51]	Y	Y	Y	Y	N	NA	Y	Y	75	Low
16	Chen et al. [52]	Y	Y	Y	Y	N	NA	Y	Y	75	Low
17	Danabal et al. [53]	Y	Y	Y	Y	N	NA	Y	Y	75	Low
18	Hossain et al. [54]	U	Y	Y	Y	U	U	Y	Y	62.5	Moderate
19	Hossain et al. [55]	Y	Y	Y	Y	U	U	Y	Y	75	Low
20	İkiışık et al. [56]	Y	Y	Y	Y	U	U	Y	Y	75	Low
21	Alabdulla et al. [57]	Y	Y	Y	Y	N	N	Y	Y	75	Low
22	Saied et al. [58]	Y	Y	Y	Y	N	N	Y	Y	75	Low
23	Qunaibi et al. [59]	N	N	N	Y	N	N	Y	Y	37.5	High
24	Faezi et al. [60]	Y	Y	Y	Y	N	N	Y	Y	75	Low
25	Milan & Dau [61]	Y	Y	Y	Y	N	N	Y	Y	75	Low
26	Allington et al. [62]	Y	Y	Y	Y	N	N	Y	Y	75	Low
27	Xu et al. [63]	Y	Y	Y	Y	Y	Y	Y	Y	100	Low

Supplement 1 *Cont.*

Sl. No.	Author(s) & Year	Q1[a]	Q2	Q3	Q4	Q5	Q6	Q7	Q8	% Yes	Risk[b]
28	Castaneda-Vasquez [64]	Y	Y	Y	Y	N	N	Y	Y	75	Low
29	Bono et al. [65]	Y	Y	Y	Y	N	N	Y	Y	75	Low
30	Al-Sanaf& Sallam [66]	Y	Y	Y	Y	N	N	Y	Y	75	Low
31	Sallam et al. [67]	Y	Y	Y	Y	N	N	Y	Y	75	Low
32	Sallam et al. [68]	Y	Y	Y	Y	N	N	Y	Y	75	Low
33	Kuçukkarapinar et al. [69]	Y	Y	Y	Y	N	N	Y	Y	75	Low
34	Plitch-loeb et al. [70]	Y	Y	Y	Y	N	N	Y	Y	75	Low
35	Alibrahim&Awad [71]	Y	Y	Y	Y	N	NA	Y	Y	75	Low
36	Acar-Burkay& Cristian [72]	N	Y	Y	Y	N	NA	Y	Y	62.5	Moderate
37	Dambadarjaa et al. [73]	Y	Y	Y	Y	N	NA	Y	Y	75	Low
38	Ebrahimi et al. [74]	Y	Y	Y	Y	N	NA	Y	Y	75	Low
39	Ehde et al. [75]	Y	Y	Y	Y	N	NA	Y	Y	75	Low
40	Almaghaslah et al. [76]	Y	Y	Y	Y	N	N	Y	Y	75	Low

Supplement 1 *Cont*

Sl. No.	Author(s) & Year	Q1[a]	Q2	Q3	Q4	Q5	Q6	Q7	Q8	% Yes	Risk[b]
41	Jain et al. [77]	Y	Y	Y	Y	U	U	Y	Y	75	Low
42	Kumar et al. [78]	Y	Y	Y	Y	N	NA	Y	Y	75	Low
43	Luk et al. [79]	Y	Y	Y	Y	N	NA	Y	Y	75	Low
44	Maraqa et al. [80]	Y	Y	Y	Y	Y	U	Y	Y	87.5	Low
45	Mejri et al. [81]	Y	Y	Y	Y	N	NA	Y	Y	75	Low
46	Navarre et al. [82]	Y	Y	Y	Y	N	NA	Y	Y	75	Low
47	Oliveira et al. [83]	Y	Y	Y	Y	U	U	Y	Y	75	Low
48	Park et al. [84]	Y	Y	Y	Y	N	NA	Y	Y	75	Low
49	Sethi et al. [85]	Y	Y	Y	Y	N	NA	Y	Y	75	Low
50	Sirikalyanpaiboon et al. [86]	Y	Y	Y	Y	N	NA	Y	Y	75	Low
51	Yahia et al. [87]	Y	Y	Y	Y	N	NA	Y	Y	75	Low
52	Yeşiltepe et al. [88]	Y	Y	Y	Y	U	U	Y	Y	75	Low
53	Albahri et al. [89]	Y	Y	Y	Y	U	U	Y	Y	75	Low
54	Singh et al. [90]	Y	Y	Y	Y	N	NA	Y	Y	75	Low

Supplement 1 *Cont.*

Sl. No.	Author(s) & Year	Q1[a]	Q2	Q3	Q4	Q5	Q6	Q7	Q8	% Yes	Risk[b]
55	Ali & Hossain [91]	Y	Y	Y	Y	U	NA	Y	Y	75	Low
56	Anjorin et al. [92]	Y	Y	Y	Y	U	U	Y	Y	75	Low
57	Boon-Itt et al. [93]	Y	Y	Y	Y	U	U	Y	Y	75	Low
58	Yilma et al. [94]	Y	Y	Y	Y	N	NA	Y	Y	75	Low
59	Fakonti et al. [95]	Y	Y	Y	Y	N	NA	Y	Y	75	Low
60	Li et al. [96]	Y	Y	Y	Y	N	NA	Y	Y	75	Low
61	Magadmi et al. [97]	Y	Y	Y	Y	U	U	Y	Y	75	Low
62	Khairat et al. [98]	Y	Y	Y	Y	N	NA	Y	Y	75	Low
63	Holeva et al. [99]	Y	Y	Y	Y	U	U	Y	Y	75	Low
64	Hubach et al. [100]	Y	Y	Y	Y	N	NA	Y	Y	75	Low
65	Lo Moro et al. [101]	Y	Y	Y	Y	U	U	Y	Y	75	Low
66	Silva et al. [102]	Y	Y	Y	Y	N	N	Y	Y	75	Low
67	Soares et al. [103]	Y	Y	Y	Y	N	N	Y	Y	75	Low

Supplement 1 *Cont.*

Sl. No.	Author(s) & Year	Q1[a]	Q2	Q3	Q4	Q5	Q6	Q7	Q8	% Yes	Risk[b]
68	Kavanagh et al. [104]	Y	Y	Y	Y	N	NA	Y	Y	75	Low
69	Hwang et al. [105]	Y	Y	Y	Y	U	U	Y	Y	75	Low
70	Hong et al. [106]	Y	Y	Y	Y	A	NA	Y	Y	75	Low
71	Shareef et al. [107]	Y	Y	Y	Y	N	NA	Y	Y	75	Low
72	Lee & You [108]	Y	Y	Y	Y	N	NA	Y	Y	75	Low
73	Kumari et al. [109]	Y	Y	Y	Y	N	NA	Y	Y	75	Low
74	Moscardino et al. [110]	Y	Y	Y	Y	U	U	Y	Y	75	Low
75	Mundagowa et al. [111]	Y	Y	Y	Y	U	U	Y	Y	75	Low
76	Zammit et al. [112]	Y	Y	Y	Y	U	U	Y	Y	75	Low
77	Ekowo et al. [113]	Y	Y	Y	Y	N	NA	Y	Y	75	Low
78	Skeens et al. [114]	Y	Y	Y	Y	U	U	Y	Y	75	Low
79	Walsh et al. [115]	Y	Y	Y	Y	N	N	Y	Y	75	Low
